# Trends in Melanoma Incidence, Prevalence, Stage at Diagnosis, and Survival: An Analysis of the United States Cancer Statistics (USCS) Database

**DOI:** 10.7759/cureus.70697

**Published:** 2024-10-02

**Authors:** Okelue E Okobi, Edelann Abreo, Nneka P Sams, Onyebuchi H Chukwuebuni, Loretta Agyemang Tweneboa Amoako, Bernard Wiredu, Emah E Uboh, Victoria C Ekechi, Adaku A Okafor

**Affiliations:** 1 Family Medicine, Medficient Health Systems, Laurel, USA; 2 Family Medicine, Lakeside Medical Center, Belle Glade, USA; 3 Family Medicine, Larkin Community Hospital Palm Springs Campus, Miami, USA; 4 Family Medicine, Grand Rehabilitation Center, Valatie, USA; 5 Family Medicine, Dr. D. Y. Patil Medical College, Hospital, and Research Centre, Mumbai, IND; 6 Public Health, Walden University, Minneapolis, USA; 7 Nursing, Walden University, Columbia, USA; 8 Internal Medicine, Spartan Health Sciences University, School of Medicine, Vieux Fort, LCA; 9 Internal Medicine, Greater Accra Regional Hospital, Accra, GHA; 10 Internal Medicine and Oncology, St. James School of Medicine, Park Ridge, USA; 11 Internal Medicine, College of Medicine, University of Lagos, Lagos, NGA; 12 Hematology and Oncology, Veterans Affairs Medical Center, Washington, DC, USA

**Keywords:** incidence, melanoma, prevalence, public health, stage at diagnosis, survival rates, uscs database

## Abstract

Background and objectives

Melanoma, a major skin cancer, has seen varying trends in incidence, prevalence, stage at diagnosis, and survival. This study examines these trends using the United States Cancer Statistics (USCS) database, covering the period from 1999 to 2021.

Methods

We extracted data from the USCS database, which integrates the National Cancer Institute’s (NCI) Surveillance, Epidemiology, and End Results (SEER) program and the Centers for Disease Control and Prevention’s (CDC) National Program of Cancer Registries (NPCR). The analysis included new melanoma cases, prevalence estimates (using a 20-year limited duration), stage at diagnosis, and five-year relative survival rates. Incidence rates were adjusted for age using the 2000 United States standard population. Descriptive and trend analyses were performed using IBM SPSS Statistics software, version 29 (IBM Corp., Armonk, NY).

Results

The analysis of melanoma trends from 1999 to 2021 reveals a significant increase in the annual age-adjusted incidence rate, rising from 15.1 per 100,000 (95% CI: 14.9- 15.2) in 1999 to 23.0 per 100,000 (95% CI: 22.8- 23.1) in 2021. This upward trend is consistent across gender and racial/ethnic groups. The prevalence of melanoma over a 20-year period was 0.279 (95% CI: 0.276-0.282), with males showing a higher prevalence (0.302, 95% CI: 0.298-0.306) compared to females (0.256, 95% CI: 0.252-0.260). The distribution of melanoma stage at diagnosis indicated that 77% of cases were localized (95% CI: 76.5-77.5%), 9.5% regional (95% CI: 9.2-9.8%), 4.7% distant (95% CI: 4.4-5.0%), and 8.8% unstaged (95% CI: 8.5-9.1%). Survival analysis showed a five-year relative survival rate of 99.4% (95% CI: 99.2-99.6%) for localized melanoma and 35.6% (95% CI: 33.7-37.6%) for distant melanoma, highlighting significant disparities in survival based on stage at diagnosis.

Conclusions

The study highlights a rising incidence of melanoma and emphasizes the critical role of early detection in improving survival outcomes. The findings underscore the effectiveness of early diagnosis and the necessity for ongoing efforts to improve melanoma outcomes across diverse populations.

## Introduction

Melanoma, a type of skin cancer originating from melanocytes, represents a significant public health challenge due to its increasing incidence and impact on survival. The burden of melanoma is characterized by its high morbidity and mortality rates, especially when diagnosed at advanced stages [1-2]. The disease burden of melanoma is substantial, with rising rates of new cases and associated mortality worldwide. Arnold's global melanoma study estimated 325,000 new cases and 57,000 deaths in 2020, with significant geographic variations, and the global burden is projected to rise to 510,000 new cases and 96,000 deaths by 2040 [3]. In the United States, melanoma is among the most common cancers, particularly affecting fair-skinned individuals, who are at a higher risk due to their lower melanin levels, which offer less protection against ultraviolet (UV) radiation [4]. Melanoma’s incidence has been steadily increasing over the past few decades, making it a critical focus for cancer research and public health strategies. According to the most recent data from the Surveillance, Epidemiology, and End Results (SEER) program, melanoma is the fifth most commonly diagnosed cancer in the United States, excluding nonmelanoma skin cancers. In 2021, it was estimated that there would be 106,000 new cases of melanoma, which represents 5.6% of all cancer diagnoses for that year [5-6]. 

The primary mechanism driving melanoma is the mutation of melanocyte cells, often induced by UV radiation from sunlight or tanning beds. These mutations lead to uncontrolled cell growth and the potential for metastasis [7]. Genetic predispositions, such as mutations in the BRAF and NRAS genes, further contribute to melanoma risk. Additionally, environmental factors and lifestyle choices, including sun exposure and tanning practices, play a significant role in the disease’s development. Understanding these mechanisms is crucial for developing effective prevention and treatment strategies [7-8]. The American Cancer Society reports that in recent years, melanoma diagnoses have increased, which can be attributed to both higher awareness and increased exposure to risk factors like UV radiation [9]. Understanding trends in melanoma incidence, prevalence, stage at diagnosis, and survival is crucial for several reasons. First, analyzing these trends provides insights into the effectiveness of public health interventions, screening programs, and advancements in treatment. Second, identifying patterns in the stage at diagnosis helps evaluate whether early detection efforts are improving over time. Third, assessing survival rates across different demographic groups can highlight disparities and inform targeted prevention and treatment strategies. This study aims to analyze trends in melanoma incidence, prevalence, stage at diagnosis, and survival using the United States Cancer Statistics (USCS) database [10]. By examining data from 1999 to 2021, we seek to identify temporal changes in these key metrics, providing insights into the effectiveness of public health interventions and the impact of advances in melanoma diagnosis and treatment.

## Materials and methods

Study design and participants' data source

This retrospective analysis employed a population-based study design, utilizing data from the USCS database to investigate trends in melanoma incidence, prevalence, stage at diagnosis, and survival. The study population comprised individuals diagnosed with invasive melanoma of the skin from 1999 to 2021. The data encompass various demographic and geographic groups across the United States. Data were sourced from the USCS database, which integrates information from the National Cancer Institute's (NCI) SEER program and the Centers for Disease Control and Prevention's (CDC) National Program of Cancer Registries (NPCR). This database provides comprehensive cancer statistics, including detailed information on incidence, prevalence, and survival across different demographic groups and geographic regions [11].

The terms "non-Hispanic," "Black non-Hispanic," "American Indian and Alaska Native non-Hispanic," and "Asian and Pacific Islander non-Hispanic" which have been used in this article as variables are obtained from the primary data source as stated. They are not the author's terms.

Inclusion and exclusion criteria

For this analysis, inclusion criteria were: 1) melanoma of the skin as the primary cancer diagnosis; and 2) availability of complete data on stage at diagnosis, survival, and demographic characteristics from the USCS database covering the years 1999 to 2021. Exclusion criteria included: 1) non-skin melanomas; and 2) cases with incomplete or missing data on key variables such as stage, survival rates, or demographic details. This approach ensured that only comprehensive and relevant data on skin melanoma were analyzed, providing accurate insights into trends in incidence, prevalence, stage at diagnosis, and survival.

Data extraction and variables, descriptive and trend analysis

Data extraction involved retrieving information on melanoma cases, including annual incidence rates, prevalence counts and percentages, stage at diagnosis, and five-year relative survival rates. Key variables included Incidence rates: the number of new melanoma cases reported annually, stratified by gender and racial/ethnic categories; Prevalence: prevalence counts and percentages, based on a 20-year limited duration approach, are categorized by gender, race, and age; Stage at diagnosis: classification into localized, regional, distant, and unstaged categories; Survival rates: five-year relative survival rates, stratified by gender, race, age group, and stage at diagnosis. Descriptive analysis was conducted using IBM SPSS Statistics software, version 29 (IBM Corp., Armonk, NY) to summarize melanoma incidence, prevalence, and survival rates using percentages and a 95% confidence interval. Frequency distributions were generated for demographic variables and stage at diagnosis. Age-adjusted incidence rates were calculated using the 2000 United States standard population to account for demographic shifts. Trends were examined across gender, racial/ethnic categories, and stages at diagnosis to identify significant patterns and changes over the study period from 1999 to 2021.

Ethical considerations

This study utilized de-identified, publicly available data from the USCS database, ensuring compliance with ethical standards for research involving human subjects. Using secondary data from established databases does not require individual consent, but adherence to ethical guidelines and proper citation of data sources were strictly observed.

## Results

Trends in melanoma incidence over time

The increasing trend in melanoma incidence underscores the need for enhanced prevention and early detection strategies (Table 1). From 1999 to 2021, the incidence of melanoma has demonstrated a clear upward trend. The age-adjusted rate for the overall population increased from 15.1 per 100,000 (95% CI: 14.9-15.2) in 1999 to 23.0 per 100,000 (95% CI: 22.8-23.1) in 2021. This represents a rise of 52.3% over the period.

**Table 1:** Annual incidence rates of new cancer cases in the United States during the study period (1999-2021)

Year	Age-adjusted rate (95% CI)	Case count	Population
1999	15.1 (14.9 - 15.2)	41,009	275,461,348
2000	16.1 (15.9 - 16.2)	44,481	278,558,214
2001	17.1 (16.9 - 17.2)	47,977	282,115,961
2002	17.4 (17.3 - 17.6)	49,830	284,766,512
2003	17.2 (17.0 - 17.3)	50,362	290,107,933
2004	18.2 (18.1 - 18.4)	54,185	292,805,298
2005	19.5 (19.3 - 19.6)	58,822	295,516,599
2006	19.3 (19.1 - 19.4)	59,205	298,379,912
2007	19.7 (19.6 - 19.9)	61,748	301,231,207
2008	20.0 (19.9 - 20.2)	63,876	304,093,966
2009	20.4 (20.3 - 20.6)	66,262	306,771,529
2010	20.1 (20.0 - 20.3)	66,497	309,382,247
2011	20.7 (20.6 - 20.9)	69,668	311,860,624
2012	20.8 (20.6 - 20.9)	71,225	314,371,446
2013	21.4 (21.3 - 21.6)	74,880	316,765,048
2014	22.3 (22.1 - 22.5)	79,443	319,294,716
2015	23.0 (22.8 - 23.1)	83,334	321,850,521
2016	23.1 (22.9 - 23.3)	85,246	324,377,907
2017	23.4 (23.2 - 23.6)	88,025	326,611,185
2018	22.8 (22.7 - 23.0)	87,446	328,525,933
2019	23.7 (23.5 - 23.8)	92,369	330,222,008
2020	20.6 (20.5 - 20.7)	79,999	324,722,713
2021	23.0 (22.8 - 23.1)	90,365	325,218,022

**Figure 1:**
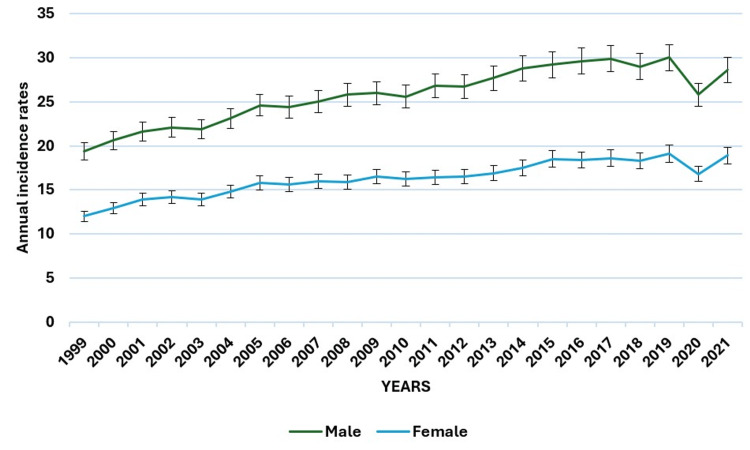
Annual age-adjusted incidence rates of melanoma by gender

The higher rates in males compared to females throughout the years highlight a gender disparity in melanoma incidence that warrants further investigation (Figure 1). For females, the age-adjusted rate rose from 12.0 per 100,000 (95% CI: 11.8-12.2) in 1999 to 18.9 per 100,000 (95% CI: 18.7-19.1) in 2021, reflecting an increase of 57.5%. In males, the rate grew from 19.4 per 100,000 (95% CI: 19.1-19.6) in 1999 to 28.6 per 100,000 (95% CI: 28.3-28.8) in 2021, a rise of 47.4%.

**Figure 2:**
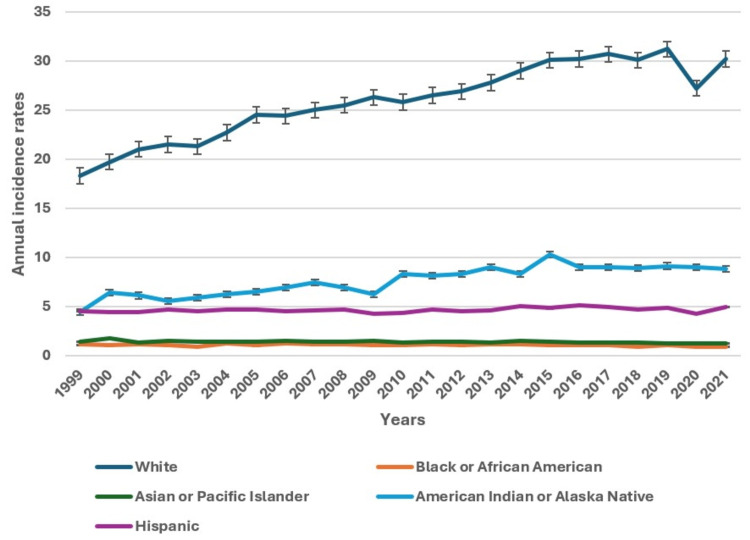
Annual age-adjusted incidence rates of melanoma by racial and ethnic categories

From 1999 to 2021, melanoma incidence rates exhibited varying trends across different racial and ethnic groups (Figure 2). For White individuals, the age-adjusted rate increased from 18.3 per 100,000 (95% CI: 18.1 - 18.5) in 1999 to 30.2 per 100,000 (95% CI: 30.0 - 30.4) in 2021, marking a rise of 64.6%. This upward trend underscores a growing incidence within this group. In contrast, Black individuals experienced a stable but low rate, starting at 1.1 per 100,000 (95% CI: 1.0-1.3) in 1999 and remaining at 0.9 per 100,000 (95% CI: 0.8-1.0) in 2021, with minimal fluctuation. Individuals who were Asian or Pacific Islanders slightly increased from 1.4 per 100,000 (95% CI: 1.2-1.7) in 1999 to 1.2 per 100,000 (95% CI: 1.1-1.4) in 2021. American Indian and Alaska Native populations saw a variable rate, peaking at 10.3 per 100,000 (95% CI: 9.0-11.7) in 2015 and decreasing slightly to 8.8 per 100,000 (95% CI: 7.7-10.1) by 2021. Hispanic individuals had a less pronounced increase, rising from 4.5 per 100,000 (95% CI: 4.2-4.8) in 1999 to 4.9 per 100,000 (95% CI: 4.6-5.1) in 2021. These trends illustrate significant disparities in melanoma incidence among racial and ethnic groups, with White individuals experiencing the most substantial increase.

Prevalence of melanoma: a 20-year limited duration analysis

The prevalence of melanoma, based on a 20-year limited duration, shows significant variation across different demographic groups (Table 2). Overall, the prevalence percentage is 0.279, corresponding to a total prevalence count of 928,191 cases. Gender disparities are evident, with males exhibiting a higher prevalence of 0.302 (498,133 cases) compared to females at 0.256 (430,058 cases). This suggests a greater burden of melanoma among men within the studied population. 

**Table 2:** Prevalence count and percentage of melanoma cases based on a 20-year limited duration The terms "non-Hispanic,"  "Black non-Hispanic,"  "American Indian and Alaska Native non-Hispanic,"  and "Asian and Pacific Islander non-Hispanic" which have been used in this article as variables are obtained from the primary data source as stated. They are not the author's terms.

Category	Variables	Prevalence percentage	Prevalence count
Total	Overall	0.279	928,191
Gender	Male	0.302	498,133
	Female	0.256	430,058
Race	White Non-Hispanic	0.448	898,940
	Black Non-Hispanic	0.007	3,222
	American Indian and Alaska Native Non-Hispanic	0.090	2,450
	Asian and Pacific Islander Non-Hispanic	0.012	2,571
	Hispanic	0.034	21,008
Age group (in years)	0-19	0.002	1,337
	20-29	0.020	9,009
	30-39	0.095	43,296
	40-49	0.203	83,531
	50-59	0.362	154,405
	60-69	0.606	237,151
	70-79	0.955	235,645
	80+	1.277	163,817

Racial differences also highlight substantial variations. White non-Hispanic individuals have the highest prevalence at 0.448 (898,940 cases), indicating a significant proportion of melanoma cases within this group. In contrast, other racial groups show much lower prevalence rates: Black non-Hispanic individuals at 0.007 (3,222 cases), American Indian and Alaska Native non-Hispanic individuals at 0.090 (2,450 cases), Asian and Pacific Islander non-Hispanic individuals at 0.012 (2,571 cases), and Hispanic individuals at 0.034 (21,008 cases). This underscores a predominant impact on White non-Hispanic populations.

Age-wise, prevalence increases significantly with age. The youngest group (0-19 years) has a minimal prevalence of 0.002 (1,337 cases), while prevalence rises steeply in older age brackets. The prevalence is 0.955 (235,645 cases) for those aged 70-79 years and peaks at 1.277 (163,817 cases) for individuals aged 80 and above. These figures reflect the cumulative risk of melanoma with advanced age, with older individuals exhibiting the highest prevalence. These trends highlight the need for targeted prevention and early detection strategies, particularly for high-risk groups such as older adults and White non-Hispanic individuals.

Stage at diagnosis and survival rates by demographic factors

The five-year relative survival rates for invasive skin melanomas in the United States reveal notable disparities based on gender, race, age, and stage at diagnosis (Table 3).

**Table 3:** Five-year relative survival rates for invasive melanomas of the skin by gender, race, and age group The terms "non-Hispanic,"  "Black non-Hispanic,"  "American Indian and Alaska Native non-Hispanic,"  and "Asian and Pacific Islander non-Hispanic" which have been used in this article as variables are obtained from the primary data source as stated. They are not the author's terms.

Category	Variables	5-year Relative Survival (%) (95% CI)
Gender	Female	95.1 (94.9 - 95.3)
	Male	92.2 (92.0 - 92.4)
Race	White, Non-Hispanic	93.2 (93.1 - 93.4)
	Black, Non-Hispanic	67.6 (64.5 - 70.5)
	American Indian and Alaska Native, Non-Hispanic	91.9 (88.7 - 94.3)
	Asian and Pacific Islander, Non-Hispanic	78.5 (75.3 - 81.3)
	Hispanic	84.7 (83.7 - 85.7)
Age Group (years)	<45	95.7 (95.5 - 95.9)
	45-54	94.4 (94.1 - 94.7)
	55-64	93.8 (93.6 - 94.1)
	65-74	93.4 (93.1 - 93.7)
	75+	91.0 (90.4 - 91.6)
	<65	94.5 (94.3 - 94.6)
	65+	92.3 (92.0 - 92.6)
Survival by Stage	Localized	99.4
	Regional	73.3
	Distant	35.6
	Unstaged	91.4

Gender

Female patients exhibit a higher five-year relative survival rate of 95.1% (95% CI: 94.9-95.3) compared to 92.2% (95% CI: 92.0-92.4) for males. This difference highlights a gender disparity in survival outcomes, with females having a slightly better prognosis.

Race

Survival rates vary significantly among racial groups. White non-Hispanic individuals have the highest survival rate at 93.2% (95% CI: 93.1-93.4). In contrast, Black non-Hispanic individuals face a much lower survival rate of 67.6% (95% CI: 64.5-70.5), indicating a pronounced racial disparity. American Indian and Alaska Native non-Hispanic individuals have a survival rate of 91.9% (95% CI: 88.7-94.3), while Asian and Pacific Islander non-Hispanic individuals and Hispanics have survival rates of 78.5% (95% CI: 75.3-81.3) and 84.7% (95% CI: 83.7-85.7), respectively.

Age

Age is a significant factor in survival rates. Younger individuals (<45 years) have a five-year relative survival rate of 95.7% (95% CI: 95.5-95.9), which declines with age. Those aged 75 and older have a survival rate of 91.0% (95% CI: 90.4-91.6), reflecting decreased survival associated with advancing age.

Stage at Diagnosis

The stage at diagnosis is the most critical determinant of survival. Survival rates are highest for localized melanoma at 99.4%, significantly lower for regional melanoma at 73.3%, and even more so for distant melanoma at 35.6%. Unstaged melanomas have a survival rate of 91.4%. Among new cancer cases, 77% are diagnosed at a localized stage, with 332,323 cases identified. Regional stage cases make up 9.5% (41,126 cases), while distant stage cases account for 4.7% (20,096 cases). Additionally, 8.8% of cases (38,149) are unstaged. This distribution underscores the prevalence of early-stage diagnoses compared to advanced stages. These results underscore the importance of early detection and stage-specific treatment strategies to improve survival outcomes, especially among higher-risk groups such as older adults and Black non-Hispanic individuals.

## Discussion

This study comprehensively analyzes melanoma trends in incidence, prevalence, stage at diagnosis, and survival. Our findings reveal several significant trends and patterns, which are discussed below in the context of existing literature. Our analysis shows a rising trend in melanoma incidence over the study period, consistent with findings from previous research [11-14]. The age-adjusted incidence rate increased from 15.1 per 100,000 in 1999 to 23.0 per 100,000 in 2021. This trend corroborates with the results of the SEER program, which also reports a steady increase in melanoma incidence rates over the past few decades [11]. The SEER program reveals a significant rise in melanoma incidence in the United States, from 13.8 to 22.6 cases per 100,000 between 1990 and 2018. This increase emphasizes the growing burden and need for better prevention and treatment. Similarly, the Global Burden of Disease study shows a global rise in melanoma incidence from 12.6 cases per 100,000 in 1990 to 17.0 in 2018, reflecting a widespread trend [11-12]. Several factors contribute to this rise, including increased exposure to UV radiation, improved diagnostic capabilities, and heightened skin cancer awareness [13]. However, our data also suggest variations across demographic groups, with higher incidence rates observed in males compared to females and among White non-Hispanic individuals compared to other racial/ethnic groups. This racial disparity aligns with previous studies that report higher melanoma incidence among lighter-skinned individuals due to reduced melanin protection against UV radiation [4, 5, 14].

This study indicates an increase in the prevalence rate, with significant variation across gender, age, and racial categories. The higher prevalence among males compared to females mirrors findings from other studies [5, 15-16]. An epidemiology study conducted by Saginala et al. reported that melanoma exhibits a particularly high prevalence among white males, with an incidence rate of 34.7 cases per 100,000 individuals. For White females, the incidence rate is somewhat lower at 22.1 cases per 100,000 [5]. These statistics underscore the significant burden of melanoma, especially within the white male population, highlighting the need for continued vigilance and targeted prevention efforts in this high-risk group [16]. In this regard, various hypotheses have been developed with the objective of explaining the higher incidence rate of melanoma in men than women. For instance, the higher incidence rate of melanoma in men has been attributed to factors that include increased tanning bed use and intermittent exposure to ultraviolet rays, which often result in sunburn, regarded as a major sun exposure type that is linked to melanoma development [16-20]. Additionally, the difference in health behavior between men and women has been attributed to the higher rates of melanoma in men than women. For example, it has been hypothesized that among them, women tend to utilize primary care more than men, leading to timely diagnosis of potential tumors in women at earlier stages, while men infrequently utilize primary care, leading to diagnosis of melanoma tumors at later stages. Consequently, women tend to be highly likely to be aware of their skin and skin conditions, as well as carry out skin self-screening and visit physicians for health-related reasons compared to men [16, 19-22].

Moreover, the racial disparities are also evident, with White non-Hispanic individuals showing a much higher prevalence than other racial groups. This is consistent with the literature highlighting the greater susceptibility of individuals with lighter skin to melanoma [17]. Age-related differences in prevalence are striking, with the highest rates observed in individuals aged 70-79 and 80+. This age distribution aligns with findings from other studies, which suggest that melanoma prevalence increases with age due to cumulative UV exposure and delayed onset of the disease [18]. The lower prevalence in younger age groups may reflect both lower cumulative UV exposure and more effective early detection and prevention measures. Our analysis of the stage at diagnosis reveals that a substantial majority of melanoma cases are diagnosed at the localized stage, with only a small percentage presenting at distant stages. This distribution reflects improvements in early detection and screening practices. However, there has been some fluctuation in the percentage of cases diagnosed at each stage over the years, which may be attributed to varying levels of public awareness and advancements in diagnostic technology. Studies have reported similar patterns, with early-stage diagnosis increasingly common due to enhanced public awareness campaigns and advancements in dermatological screening [19-20]. Despite this, the percentage of cases diagnosed at later stages remains a concern, indicating the need for continued focus on preventive measures and early detection strategies. The five-year relative survival rates for melanoma demonstrate overall high survival rates. These figures are comparable to those reported in previous studies, which consistently show high survival rates for early-stage melanoma [21-22]. Our survival rates align with findings from a study conducted by, where survival rates for localized melanoma are exceptionally high, while those for regional and distant stages are considerably lower [22].

Strengths and limitations, implications, and future directions

This study leverages a robust dataset from the USCS database, integrating data from the SEER program and NPCR, ensuring comprehensive coverage and representativeness. The longitudinal nature of the data, spanning from 1999 to 2021, allows for a detailed analysis of trends over two decades. Using age-adjusted incidence rates and prevalence percentages clarifies trend analysis and comparison across demographic groups. Additionally, including survival data stratified by stage, gender, race, and age offers valuable insights into the effectiveness of early detection and treatment strategies. Despite its strengths, the study has limitations. The reliance on secondary data means that inaccuracies in the original data reporting could affect results. There may be incomplete data on staging or survival for some cases, particularly for the unstaged category. Additionally, variations in data collection practices across different registries could introduce inconsistencies. The study does not account for potential changes in diagnostic practices or treatment advancements during the study period.

The rising incidence and stable high survival rates for localized melanoma underscore the importance of continued efforts in prevention, early detection, and public awareness. Public health campaigns should focus on enhancing sun protection behaviors, especially among high-risk populations, and improving access to regular skin examinations. The disparities observed in incidence and survival rates across gender and racial/ethnic groups highlight the need for targeted interventions to address these inequalities. Future research should explore the underlying factors contributing to these disparities and evaluate the effectiveness of tailored prevention and treatment strategies.

## Conclusions

This study provides a comprehensive analysis of melanoma trends in incidence, prevalence, stage at diagnosis, and survival using the USCS database. It highlights the increasing incidence of melanoma, with significant disparities across gender and racial/ethnic groups. The high survival rates for localized melanoma emphasize the importance of early detection and intervention. However, the persistence of late-stage diagnoses and survival disparities underscores the need for continued public health efforts. Future research should address these disparities and evaluate the impact of new prevention and treatment strategies. Overall, the findings contribute to a better understanding of melanoma epidemiology and inform strategies for improving melanoma outcomes.
